# Gas-Phase Formation of Highly Luminescent 2D GaSe Nanoparticle Ensembles in a Nonequilibrium Laser Ablation Process

**DOI:** 10.3390/nano10050908

**Published:** 2020-05-08

**Authors:** Salah Elafandi, Zabihollah Ahmadi, Nurul Azam, Masoud Mahjouri-Samani

**Affiliations:** Department of Electrical and Computer Engineering, Auburn University, Auburn, AL 36849, USA; sge0008@auburn.edu (S.E.); zza0017@auburn.edu (Z.A.); mna0018@auburn.edu (N.A.)

**Keywords:** 2D materials, 2D nanoparticles, 2D quantum dots, laser ablation, laser-based synthesis

## Abstract

Interest in layered two-dimensional (2D) materials has been escalating rapidly over the past few decades due to their promising optoelectronic and photonic properties emerging from their atomically thin 2D structural confinements. When these 2D materials are further confined in lateral dimensions toward zero-dimensional (0D) structures, 2D nanoparticles and quantum dots with new properties can be formed. Here, we report a nonequilibrium gas-phase synthesis method for the stoichiometric formation of gallium selenide (GaSe) nanoparticles ensembles that can potentially serve as quantum dots. We show that the laser ablation of a target in an argon background gas condenses the laser-generated plume, resulting in the formation of metastable nanoparticles in the gas phase. The deposition of these nanoparticles onto the substrate results in the formation of nanoparticle ensembles, which are then post-processed to crystallize or sinter the nanoparticles. The effects of background gas pressures, in addition to crystallization/sintering temperatures, are systematically studied. Scanning electron microscopy (SEM), transmission electron microscopy (TEM), photoluminescence (PL) spectroscopy, and time-correlated single-photon counting (TCSPC) measurements are used to study the correlations between growth parameters, morphology, and optical properties of the fabricated 2D nanoparticle ensembles.

## 1. Introduction

During the past decade, a large family of two-dimensional (2D) materials beyond graphene have been under intense investigation [1,2,3]. Examples of such 2D layered structures include hexagonal boron nitride (hBN) [4], metal chalcogenides (MCs: e.g., GaSe, InS) [5] and transition metal dichalcogenides (TMDCs: e.g., MoS_2_, WSe_2_) [6,7]. These 2D materials family offer a broad range of remarkable electrical [7,8,9], optical [10], chemical [11], and mechanical properties [12] that are often originated from their structural and quantum confinement to the 2D plane [13,14,15]. In general, 2D materials are an appealing group of materials to substitute or complement traditional 3D electronic and optoelectronic materials [16,17,18].

When these 2D materials are additionally confined in the lateral dimensions, zero-dimensional (0D) nanoparticles can be formed, mimicking the potential properties of quantum dots (QDs) [19,20,21]. These 2D nanoparticles show improved or new properties in addition to the inherent properties of their parent 2D materials [22,23]. Low toxicity [24], higher specific surface area [25,26], tunable luminescence [27,28,29], improved dispersibility in both aqueous and nonaqueous solvents [30,31], ability to hybridize with other nanomaterials [32,33], in addition to doping and functionalization flexibility [34,35] are a few of the advantages exhibited by such 2D nanoparticles. Therefore, they are strong candidates for electronic [36], optical [33], energy [4], biomedical [28], sensing [23], and catalytic [37] applications. Ultrasonication-based [38,39], especially the ones accompanied by solvothermal treatments [40,41], syntheses methods have been widely adopted to produce 2D nanoparticles due to their low toxicity and ability to maintain the intrinsic properties of 2D bulk crystals [42]. However, these techniques are time-consuming with low quantum yield, production yield, and repeatability [42]. To address such lengthy processes, femtosecond laser ablation in the aqueous environment has been introduced [43,44], which shows great promise as a fast and green approach to fabricate and functionalize 2D QDs [35,45]. Another effective method to obtain large-scale monolayer QDs is intercalation-assisted exfoliation [36,46]. However, this method could lead to phase transitions [47] and contaminations [48]. Electrochemical synthesis [49,50] is another low-cost technique but with better reproducibility. In general, these methods lack compositional tunability as well as compatibility with the direct deposition and digital formation of hybrid materials and heterostructures.

Among 2D materials, gallium selenide (GaSe) is a direct bandgap material (approximately 2.2 eV) in its bulk form that has D_3h_ symmetry with a lattice constant of 0.374 nm [51]. In 1996, Stoll et al. obtained “strings of pearls”-shaped GaSe nanoparticles with a mean diameter of 42 nm through the metal–organic chemical vapor deposition (MOCVD) synthesis process [52]. The colloidal GaSe was obtained a year later by Allakhverdiev et al. through the ultrasonication of bulk GaSe crystal in methanol [53]. Moreover, in 2001, Chikan and Kelly obtained highly confined and luminescent surface-capped GaSe nanoparticles using high-temperature inorganic synthesis and column chromatography [54]. In recent years, the high-pressure pulsed laser deposition (PLD) process has shown the ability to form metastable nanoparticles in the gas phase [55]. For instance, Mahjouri-Samani et al. have recently reported the formation of various metastable nanoparticle and nanosheets using a high-pressure PLD process [56]. Dai et al. have also reported the deposition of CdSe QDs on Zn_2_SnO_4_ nanowires by PLD [57].

Here, we report a solution-free, fast, and effective laser-based approach to synthesize highly luminescent 2D GaSe nanoparticle ensembles. The pulsed laser ablation/deposition (PLA/PLD) method is used as a versatile method to ablate a bulk GaSe target and form a stoichiometric plume (see Supporting Information). Condensation of this plume in background argon gas pressure allows tuning the formation of aggregates and nanoparticles in the gas phase. Our approach simplifies the complexity of existing methods through the elimination of slow and uncontrolled chemical reactions. In addition, this method has the potential of forming tunable nanoparticles heterostructures by alternating the ablation target during the deposition. Scanning electron microscopy (SEM), transmission electron microscopy (TEM), photoluminescence (PL) spectroscopy, and time-correlated single-photon counting (TCSPC) spectroscopy were used to study the correlations between the growth parameters, morphology, and optical properties of fabricated structures.

## 2. Materials and Methods

The pulsed laser ablation/deposition experiments in this study were performed in a 21-inch spherical vacuum chamber. Si/SiO_2_ substrates (2 × 2 cm) were placed at the tip of the plume and parallel to the target. An excimer laser (CompexPro KrF 248 nm wavelength with 20 ns pulse duration, Coherent Inc., Santa Clara, CA, USA) was used to ablate a rotating bulk GaSe target in order to generate GaSe nanoparticles and deposit them onto the Si/SiO_2_ substrates. The target was irradiated at a 45° angle of incidence with repetition rates of 2 Hz. The laser repetition rate of 2 Hz was chosen to allow enough time for the generated plume to clear before the next plume arrives in order to minimize the effect of plume–plume collisions. Laser energy of 300 mJ with a 2 × 5 mm beam size (i.e., 3 J·cm^−2^) on the target was used to ensure the stoichiometric transfer of materials. The substrate to target distance was adjusted to be a few millimeters above the visible laser plume to ensure the collection of pure nanoparticles on the substrates. The deposition was up to 5000 pulses to collect an adequate amount of nanoparticles for subsequent characterizations.

The ensemble nanoparticles were heat-treated using a 3-inch diameter 3-zone tube furnace. The nanoparticle deposited on the Si/SiO_2_ substrates were placed inside a ceramic boat and entered into the center zone of the tube furnace. The tube was first pumped down to a few millitorrs. Before heating, the pressure was increased to atmospheric pressure by flowing an argon gas into the tube. During the heating process, 100 sccm Ar gas was continuously flowing through the quartz tube. The samples were treated under various temperatures ranging from 200 to 500 °C. After heating, the furnace was turned off, and the substrates were cooled down to room temperature while the Ar gas was flowing to avoid oxidation.

Photoluminescence (PL) spectroscopy and lifetime measurements were performed in a custom-made optical spectroscopy system capable of measuring PL and PL lifetime. The PL measurements were performed using a 50× objective lens (NA = 0.75, HORIBA Scientific, Piscataway, NJ, USA). A Horiba HR spectrometer (HORIBA Scientific, Piscataway, NJ, USA) with a 300 g/mm grating was used for PL. A picosecond 405 nm laser and a continuous-wave 532 nm laser were used as excitation sources. The laser power was minimized to avoid potential beam-induced alteration of the nanoparticles during the measurements. Lifetime measurements were performed using a Horiba TCSPC system (HORIBA Scientific, Piscataway, NJ, USA) with a picosecond 405 nm laser as the excitation source. The number of counts was limited to 1000 counts in order to avoid potential beam-induced damage or alteration of the nanoparticles. Horiba EzTime Software (HORIBA Scientific, Piscataway, NJ, USA) was used to collect and analyze the lifetime measurement data.

A high-resolution Zeiss EVO 50 variable pressure Scanning Electron Microscopy (SEM) (Carl Zeiss Microscopy LLC, White Plains, NY, USA) attached to Oxford Instruments INCA spectrometer for energy-dispersive X-ray spectroscopy (EDX) (Oxford Instruments NanoAnalysis, Concord, MA, USA) was used to characterize the morphology and structural composition of the deposited nanoparticles. SEM images were obtained with 10 kV accelerating voltage, while EDX was performed using 20 kV. The EDX data were analyzed using Oxford INCA software (Oxford Instruments NanoAnalysis, Concord, MA, USA). For transmission electron microscopy (TEM) imaging, a Zeiss EM10 transmission electron microscope (Carl Zeiss Microscopy LLC, White Plains, NY, USA) with an accelerating voltage of 60 kV was used for single-particle and aggregation analysis. The TEM grids were prepared by first sonicating the nanoparticles in ethanol for 1 min, followed by steering the TEM grids inside the solution to collect the nanoparticles.

## 3. Results and Discussion

**Figure 1:**
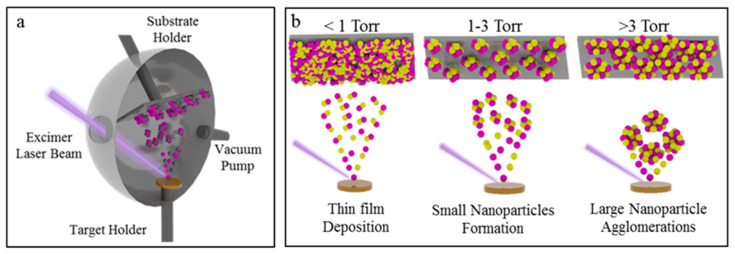
Schematic illustration of the experimental setup used for the formation and assembly of the 2D nanoparticles in this study (**a**). Schematic illustration of the laser-generated plume dynamic and evolution of nanoparticle formation as a function of background pressure (**b**).

Typically, the PLD process (Figure 1a) involves the formation of a forward-directed laser-generated plasma consisting of fast ions and neutral atoms followed by slower-moving molecules and clusters [51]. For instance, the ablation of a target in a vacuum results in the formation of fast ions and neutrals with sufficiently high kinetic energies that can form dense films when deposited on a substrate. However, background gas pressures can be used to condense the laser-generated plume, resulting in the formation of nanoparticles in the gas-phase. Figure 1b shows the schematic illustration of the plume dynamic as a function of background gas pressures, while other parameters (e.g., laser fluence, repetition rate) are kept constants.

**Figure 2:**
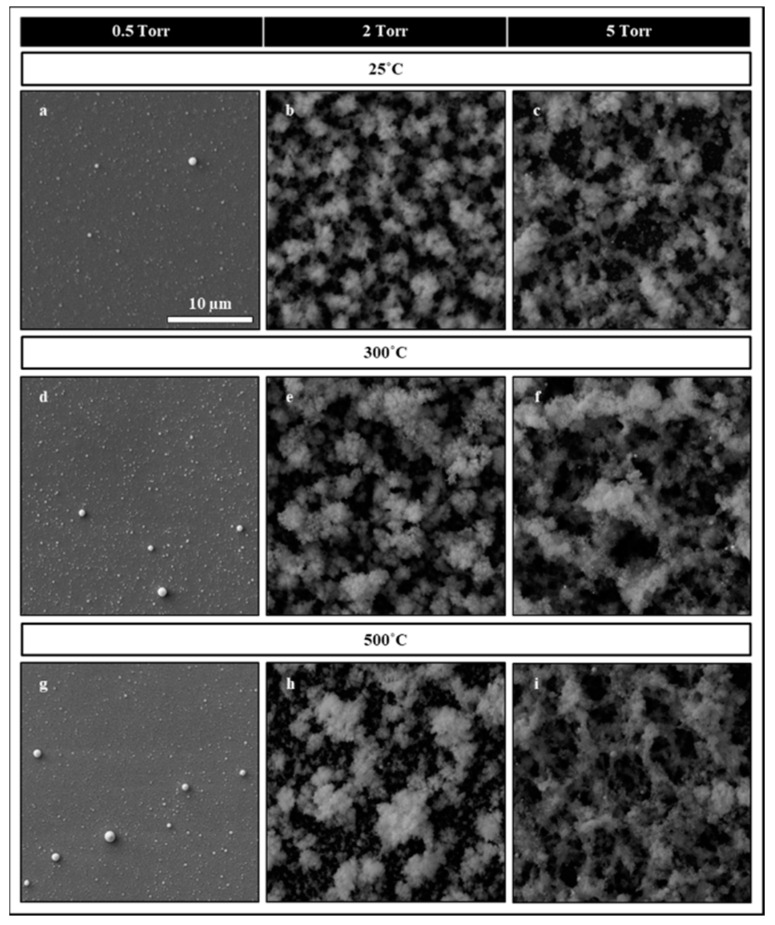
Scanning Electron Microscopy (SEM) Images of ensemble nanoparticles deposited at 0.5 (**a**,**d**,**g**), 2 (**b**,**e**,**h**), and 5 torr (**c**,**f**,**i**). The baking temperature effects on the morphology of the nanoparticles ensembles for different pressure conditions are shown for room temperature (**a**–**c**), 300 °C (**d**–**f**), and 500 °C (**g**–**i**). The deposition morphology shows a denser film at 0.5 torr and becomes more mesoporous at higher pressures. Temperature treatments of the samples resulted in the crystallization and sintering of the nanoparticles and the formation of a larger blub of nanoparticles. All images are on the same scale bar.

To tune the plume condensation dynamics for the formation of nanoparticles, argon gas was used to adjust the background pressure ranging from 0.5 to 5 torr. This pressure range allowed us to create depositions ranging from dense film to mesoporous structures as a function of increasing pressure. SEM images of room temperature-deposited structures at different background pressures are shown in Figure 2a–c. The images revealed that at pressures up to 0.5 torr (Figure 2a), mainly dense films are formed on the substrates due to the existence of atoms, molecules, and smaller aggregates in the plume. As the pressure was increased to around 2 torr (Figure 2b), the plume condensed to a semi-sphere of about 5 cm in diameter. In this condition, nanoparticles mainly started forming and creating mesoporous structures, as deposited onto the substrate. Increasing the pressure close to 5 torr (Figure 2c) resulted in the intense condensation of the plume to a semi-sphere of about 1.5 cm in diameter. This high condensation created a partially crystallized and sintered agglomeration of nanoparticles, which created loosely connected and fluffier structures when deposited onto the substrate. It should be noted that pressures beyond 5 torr resulted in small plume sizes that were challenging to bring the substrate close by for deposition. In general, the density and size of the nanoparticle agglomerations were found to be in direct correlation with background pressure due to the condensation effect induced by the background pressure.

Following the deposition, nanoparticles were baked at different temperatures aiming reduction of surface traps and studying their behavior under different temperatures. At atmospheric background pressure, a temperature window of 200 to 500 °C was used for baking the nanoparticles in a 3-zone tube furnace for 30 min with continuous argon flow throughout the baking and cooling process to avoid oxidations. The SEM images of nanoparticles deposited at 0.5, 2, and 5 torr and heat-treated at 300 °C and 500 °C are shown in Figure 2d–i. The SEM images revealed that as the temperature increased, the nanoparticles start sintering together, forming larger agglomerates and pores, as clearly seen in the samples deposited at 2 and 5 torr background pressures. The depositions at 0.5 torr (Figure 2d,g) are nearly continuous dense structures, and their sintering does not reveal significant morphological changes.

**Figure 3:**
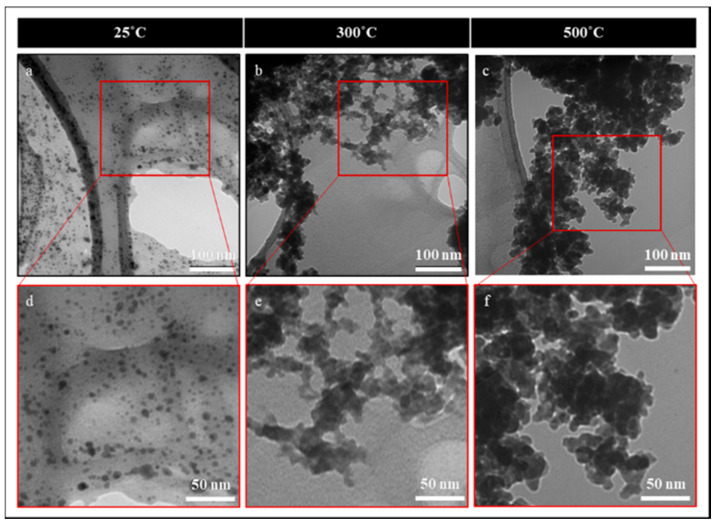
Transmission electron microscopy (TEM) images of the nanoparticles deposited at 2 torr in the as-deposited case (**a**,**d**) in addition to the baked cases at 300 °C (**b**,**c**) and 500 °C (**c**,**f**). The TEM images in the as-deposited case are well separated. However, the particles are sintering at higher temperatures.

As shown in Figure 3, TEM imaging was used to directly observe the nanoparticle size and structural evolution of nanoparticles deposited at 2 torr and under different crystallization temperatures. Samples were sonicated in ethanol and captured onto TEM grids for imaging. The room temperature-deposited samples were easily separated from each other, and individual nanoparticles of about 5–10 nm in size were collected on the TEM grids (Figure 3a,d). Partial crystallization and the sintering of nanoparticles was observed for the samples heat-treated at 300 °C (Figure 3b,e). At 500 °C (Figure 3c,f), the further sintering of nanoparticles into larger structures was clearly observed. These observations were consistent with the morphological evolutions observed in the SEM images.

**Figure 4:**
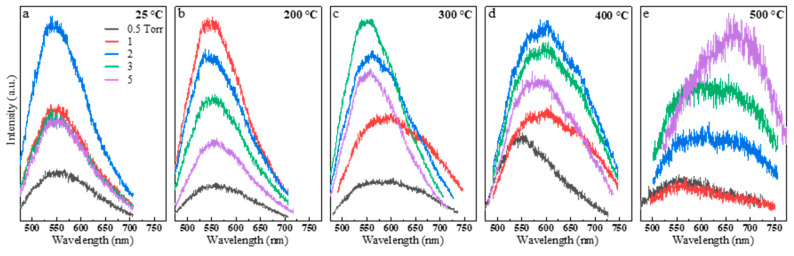
Photoluminescence (PL) spectra of the nanoparticles deposited at various pressure for the as-deposited case (**a**) and at the indicated baking temperatures (**b**–**e**). The sample deposited at 0.5 torr shows the weakest PL emission for all due to the formation of dense films (i.e., no nanoparticles). The PL spectra at 2, 3, and 5 torr have the maximum intensities due to the formation of nanoparticles.

**Figure 5:**
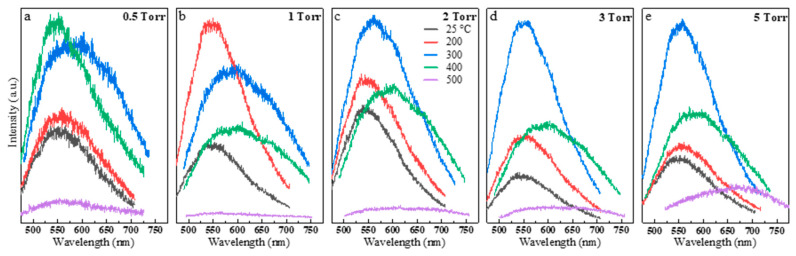
PL spectra showing the effect of baking temperature on the nanoparticles deposited at the indicated pressures (**a**–**e**). As the temperate rises to a suitable baking temperature of approximately 200–300 °C, the nanoparticles emission increases largely, and at higher temperatures (i.e., 500 °C) the intensity reduces again due to sintering and formation of larger particles.

The optical properties of the nanoparticle ensembles were studied to understand the correlation between the PL emission, deposition pressures, and crystallization temperatures. The PL spectroscopy was performed using a 405 nm picosecond laser as an excitation source. The laser power was minimized to avoid any photo-induced damage, crystallization, sintering, or oxidation of the nanoparticles during the optical spectroscopy measurements. Figure 4a–e shows the PL emission of the ensemble nanoparticles formed at different background pressures (0.5, 1, 2, 3, and 5 torr) in the as-deposited case and at the indicated crystallization/sintering temperatures (200, 300, 400, and 500 °C for 30 min). For the ease of observation, these data are also replotted in Figure 5a–e to show the crystallization/sintering temperature effect at 0.5, 1, 2, 3, and 5 torr. The PL spectra were also obtained and analyzed using a continuous-wave 532 nm laser (see Supporting Information).

Three interesting phenomena were observed while studying the effect of pressures and temperatures on the PL properties of the deposited nanoparticles. First, we observed a strong PL emission from the room temperature-deposited amorphous nanoparticle (Figure 4a), exhibiting a significant blue-shifted emission of approximately 540 nm compared to the bulk GaSe crystal central emission of approximately 625 nm (for more information about central emission and full width at half maximum (FWHM) values, check Tables S1 and S2). In these room temperature-deposited samples, the PL emission of the samples at 0.5 torr had the weakest intensity of all due to the formation of a dense film on the substrate. The PL intensity increased for nanoparticles deposited at higher pressures, with 2 torr exhibiting the maximum intensity. Second, we observed strong PL enhancements with minimal FWHM widenings by increasing the baking temperatures up to 300 °C (Figure 4b,c). At higher temperatures (Figure 4d,e), the PL intensity was then severely dropped, the FWHM was broadened, and the central emission red-shifted toward the emission of bulk crystal and beyond. Third, the lower pressure deposited samples experienced red-shifting and broadening at a lower temperature than high pressure-deposited samples. For instance, at 200 °C (Figure 4b), the samples deposited at 1 and 2 torr showed the highest intensity values compared to other pressures. In addition, they had the most blue-shifted emissions and lowest FWHM values. At 300 °C (Figure 4c), the nanoparticles deposited at 2, 3, and 5 torr showed higher intensities, minimal broadening, and red-shift, while 0.5 and 1 torr ensembles witnessed significant broadening and red-shifting. At 400 and 500 °C (Figure 4d,e), significant emission broadening, red-shift, and a reduction in the intensities are observed (as seen from the noisy spectra). The low pressure deposited samples (i.e., 0.5 and 1 torr), which were more like thin films rather than nanoparticles, appeared unstable at higher temperatures (e.g., 400–500 °C) as they revealed random changes in their optical properties.

To better observe the effect of baking temperatures on the PL emission characteristics, the temperature effects were plotted for nanoparticles deposited at each background pressure (Figure 5). For 2, 3, and 5 torr samples (Figure 5c–e), a slight red-shift is observed. However, the intensity continued increasing, reaching its maximum at approximately 300 °C, but this was followed by the steady dropping of the intensity values at higher temperatures. The intensity enhancement suggests that heat treatments lead to crystallization of nanoparticles and reduction of defects. The FWHM’s slight widening and the red-shifting effect could be due to the formation of larger particles as they slowly sinter together at low temperatures (i.e., 200 and 300 °C). However, the sintering effect at the higher temperatures (i.e., 400 and 500 °C) could create nanoparticles with random size distributions as well as degradation and formation of defects in the structures. Therefore, such significant broadening and red-shifting are clearly seen at higher baking temperatures. Such analyses are still primary, and further investigations are needed to fully understand the fundamental mechanisms governing such behaviors.

**Figure 6:**
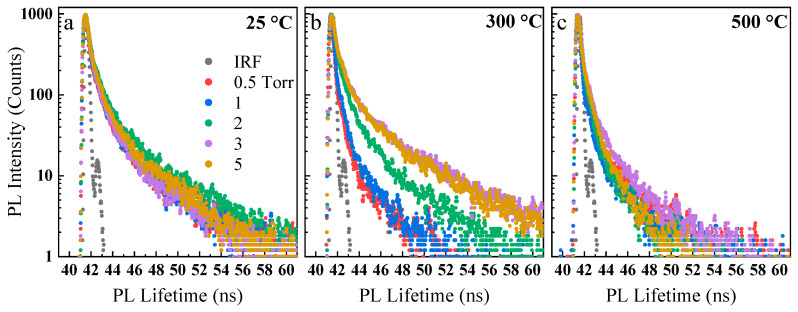
PL lifetime of the nanoparticles deposited at various pressure and heat-treated at different temperatures, including the as-deposited (**a**), 300 °C (**b**), and 500 °C (**c**). The overall trend shows that the PL lifetime increases as the deposition pressure increases.

The PL lifetime of the synthesized nanoparticles was obtained using a time-correlated single-photon counting (TCSPC) technique. The measurements were performed using a picosecond 405 nm excitation source. The curves were fitted using EzTime software (HORIBA Scientific, Piscataway, NJ, USA) and tri-exponential function and characteristic lifetimes were obtained (see Supporting Information). In general, the lifetime of all samples was less than 0.4 ns compared to the 1 ns lifetime of the bulk GaSe crystal. According to the measurements, the average lifetime of the room temperature-deposited samples (Figure 6a) gradually increased up to 2 torr and then slightly decreased afterward. At 300 °C (Figure 6b), lifetime was found to be positively correlated to the deposition background pressure. In general, it could be inferred that the partial crystallization of nanoparticles and hence reduction of the defects are occurring inside the laser-generated plasma at higher deposition pressures. As for the nanoparticles baked at 500 °C (Figure 6c), the lifetimes decreased again due to possible defect formation at this temperature, similar to the PL emission behaviors.

**Figure 7:**
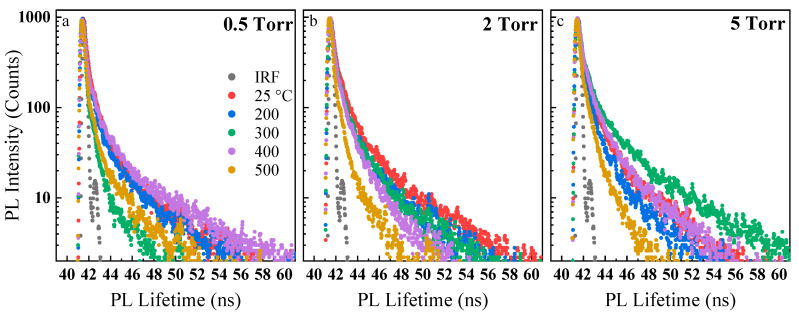
PL lifetime showing the effect of baking temperature on the nanoparticles deposited at the indicated pressure (**a**–**c**). The overall trend shows that the PL lifetime decreases as the baking temperature increases.

To better observe the effect of baking temperatures on the PL lifetime of the samples, the temperature effects were plotted for nanoparticles deposited at each background pressure (e.g., 0.5, 2, and 5 torr) (Figure 7). For instance, the average lifetime of the samples deposited at 2 torr (Figure 7b) noticeably reduced from 0.388 ns for the room temperature-deposited nanoparticles to 0.29 ns at 200 °C. The average lifetime was almost equal for 200 °C and 300 °C and then decreased slowly to 0.138 ns when baked at 500 °C. This agrees well with the red-shift and broadening of 2 torr PL, indicating increased crystallization and moderate sintering of the nanoparticles at low baking temperatures and the formation of new defects as the temperature increases. For the sample deposited at 0.5 torr (Figure 7a), the average lifetime decreased up to 300 °C, which was similar to the 2 torr sample. However, it increased again at approximately 400 °C. For the samples deposited at 5 torr (Figure 7c), the average lifetime decreased from 0.374 ns for the room temperature-deposited nanoparticles to 0.266 ns for the nanoparticles baked at 200 °C. The lifetime then increased again at approximately 300 °C, and it gradually reduced up to 500 °C. This behavior was again in agreement with our previous PL emission behavior and analyses. In general, the samples tend to crystalize and minimize their defect density when baked up to certain temperatures (i.e., approximately 300 °C), and at higher temperatures (i.e., 400 and 500 °C), new temperature-induced degradation and defects are further formed. This model is similar to the model proposed by Fassl et al. [58] on MAPbI_3_ perovskite films. Such initial room-temperature analyses open the way for more studies on the effect of surface and deep defects on optical properties of GaSe and other 2D nanoparticles in solution-less conditions.

**Figure 8:**
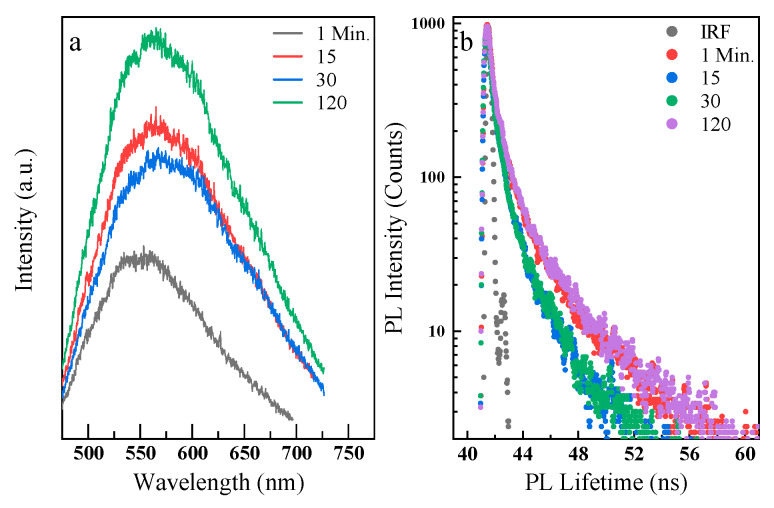
Baking time effect on the nanoparticles deposited at 2 torr and baked at 300 °C. PL spectra increase as the baking time increases (**a**). This is possibly due to defects reduction. The average PL lifetime decreases up to 30 min baking time and then increases at 120 min (**b**).

According to the PL emission and lifetime results, samples prepared at 2 torr background pressure showed the optimum optical properties. The crystallization of these nanoparticles at 200–300 °C significantly increased their emission intensity with minimal FWHM broadening. Therefore, 2 torr nanoparticles were heated at 300 °C for 1, 15, 30, and 120 min to understand the effect of baking time on PL emission and PL lifetime of the nanoparticles. A significant increase in the PL intensity accompanied by a gradual broadening was observed by increasing the baking time (Figure 8a). In addition, the average PL lifetime decreased from about 0.4 ns for 1 min to 0.25 ns for 30 min of baking time, respectively (Figure 8b). However, with longer baking times (i.e., 120 min), the lifetime increased to 0.4 ns. This outcome confirms the competition between crystallization and sintering during the heat treatments, as also seen in the above PL data.

## 4. Conclusions

In summary, 2D GaSe nanoparticle ensembles mimicking the quantum dots behaviors can be formed in the gas phase by precisely tuning the nonequilibrium environment in the laser ablation process. The room temperature deposition of these nanoparticles results in the formation of amorphous nanoparticle ensembles on the substrates that can be post-crystallized or sintered. A strong PL can be observed from the amorphous nanoparticles deposited at background pressures. As the baking temperature increases, the nanoparticles tend to crystallize and reduce their defects, leading to the enhanced PL intensities and longer lifetimes. However, increasing the temperatures beyond a threshold results in sintering these nanoparticles together, forming bigger structures, generating new defects, or inducing a phase change that could alter the PL emission intensities, central frequency, and lifetime. This nonequilibrium gas-phase method allows us to investigate the formation of other 2D nanoparticles and explore the new properties emerging from such 2D quantum dot-like structures. In addition, this method allows the formation of designed heterostructures among various 2D nanoparticles.

## Supplementary Materials

The following are available online at https://www.mdpi.com/2079-4991/10/5/908/s1, Figure S1: Energy Dispersive X-ray (EDX) analysis, Figures S2 and S3: PL spectra of the nanoparticles deposited at various pressures and baking temperatures using a 532 nm continuous-wave laser, Figures S4 and S5: PL lifetime of the nanoparticles deposited at various pressures and temperatures, Figure S6: PL lifetime and PL spectra of a bulk GaSe crystal. Tables S1–S4: Pl central emission and FHWM values, Tables S5–S9: Lifetime fitting parameters.
