# Supplementary material for: Gas-Phase Formation of Highly Luminescent 2D GaSe Nanoparticle Ensembles in a Nonequilibrium Laser Ablation Process
Source: Nanomaterials (Basel). 2020 May 8;10(5):908. doi: 10.3390/nano10050908 (PMC7279401; doi:10.3390/nano10050908)
Supplement: Supplementary file 1 [file nanomaterials-10-00908-s001.pdf]

Supporting Information

Gas-Phase Formation of Highly Luminescent 2D GaSe Nanoparticle Ensembles in a Nonequilibrium Laser Ablation Process

Salah Elafandi[1], Zabihollah Ahmadi[1], Nurul Azam[1], and Masoud Mahjouri-Samani[1*]
[1]Department of Electrical and Computer Engineering, Auburn University, Auburn, AL, USA
*Address correspondence to mahjouri@auburn.edu

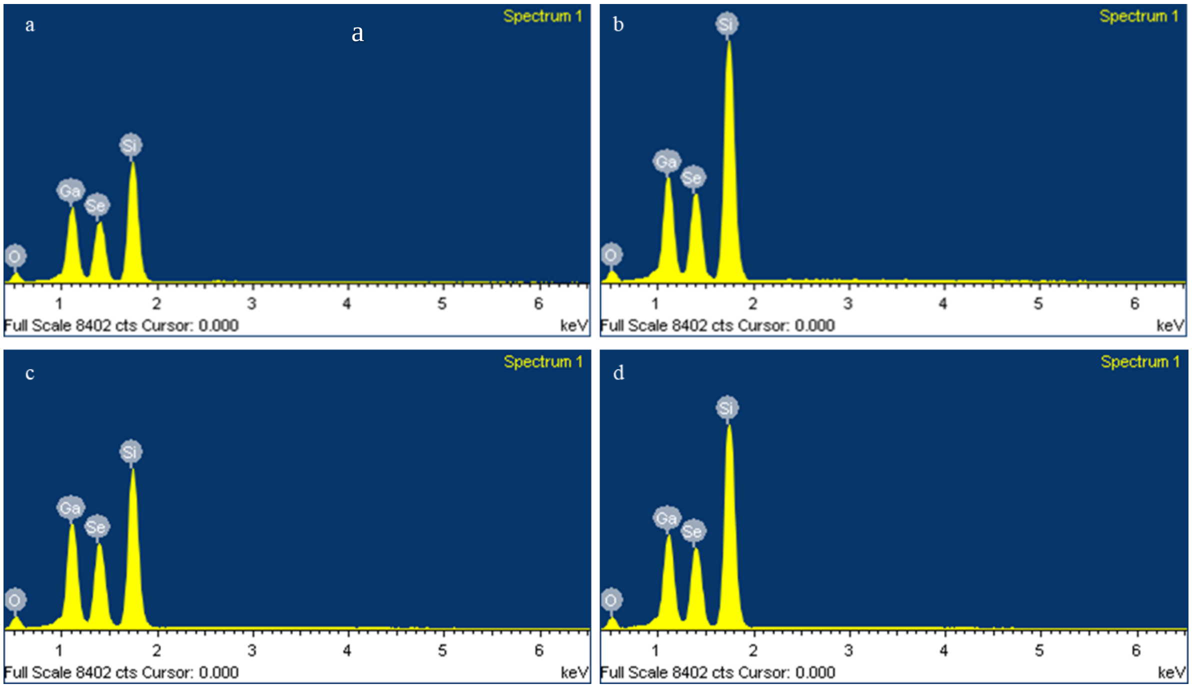

Figure S1. Energy Dispersive X-ray (EDX) results showing the stoichiometry of the as-deposited nanoparticles at 2 torr (**a**) and 5 torr (**b**) background pressures. The results verify that the stoichiometry is maintained even after baking the sample at 400ºC (**c,d**).

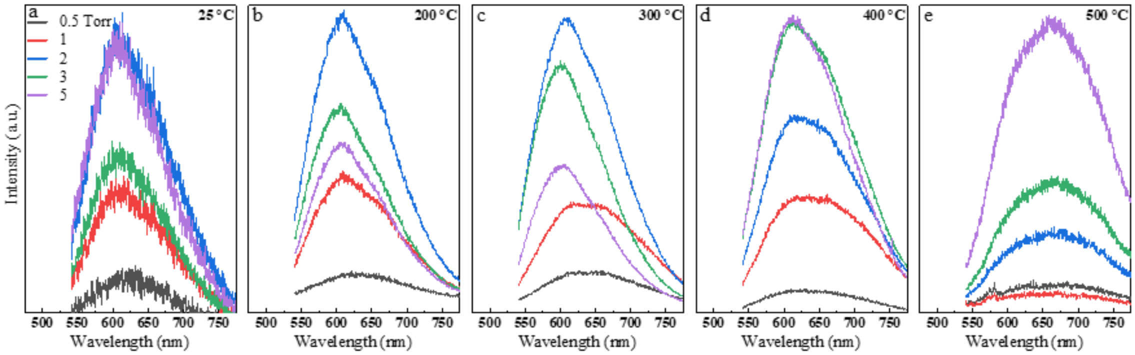

Figure S2. PL spectra of the nanoparticles deposited at various pressures and temperatures (**a-e**), using a 532 nm continuous-wave laser. The sample deposited at 0.5 torr shows the weakest PL emission for all temperatures due to the formation of dense films (i.e., no nanoparticles). The PL spectra of samples deposited at 2, 3 and 5 torr have the maximum intensities due to the formation of nanoparticles.

Supporting Information

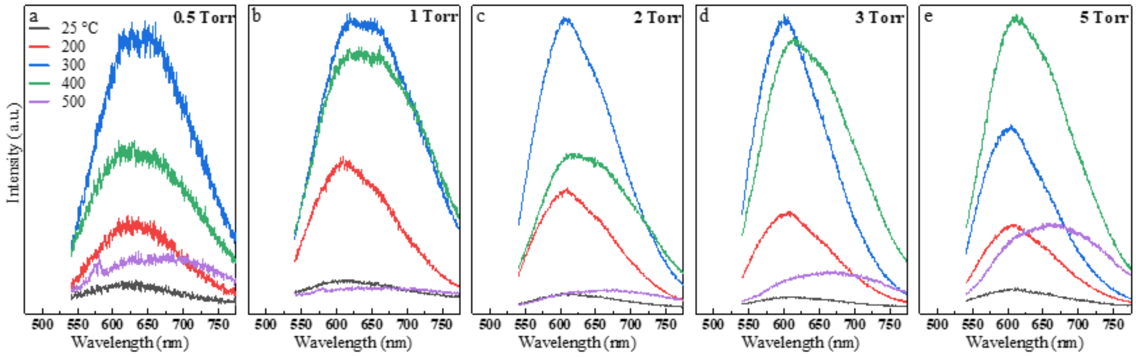

Figure S3. PL spectra showing the effect of baking temperature on the nanoparticles deposited at the indicated pressure (**a-e**), using a 532 nm continuous-wave laser. Room temperature deposited samples show the weakest emission. As the temperate increases to a suitable crystallization temperature of ~300 °C, nanoparticles emission increases largely, and at higher temperatures (i.e., 500 °C) the intensity reduces again due to sintering and formation of larger particles.

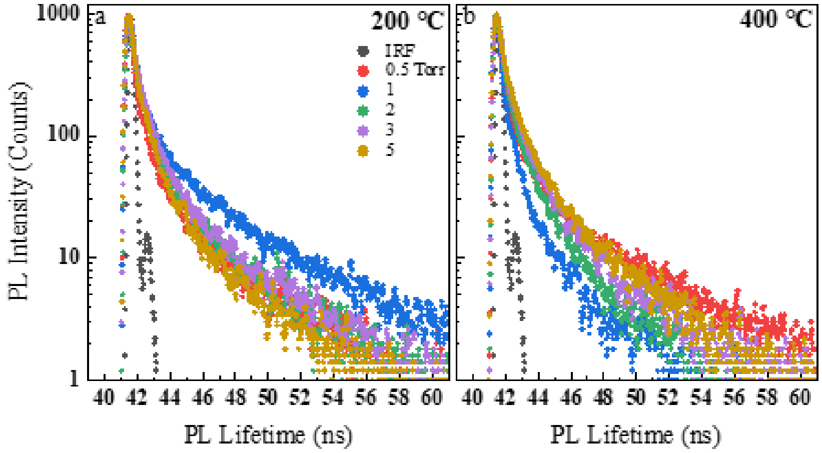

Figure S4. PL lifetime of the nanoparticles deposited at various pressures banked at 200 (**a**) and 400 ºC (**b**).

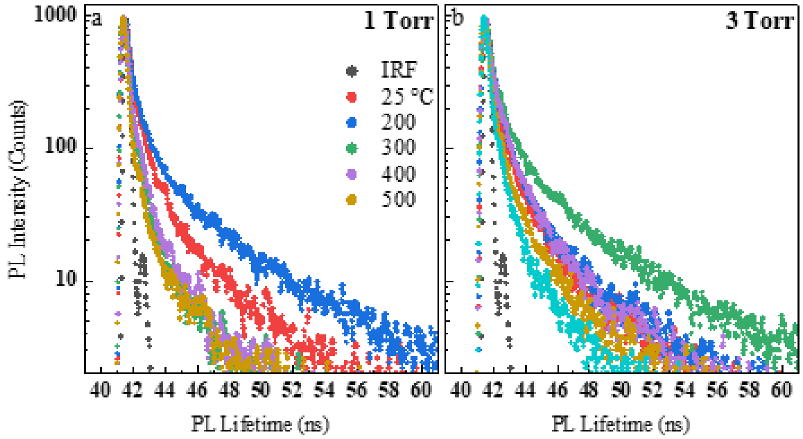

Figure S5. PL lifetime showing the effect of baking temperature on the nanoparticles deposited at 1 (**a**) and 3 (**b**) torr.

Supporting Information

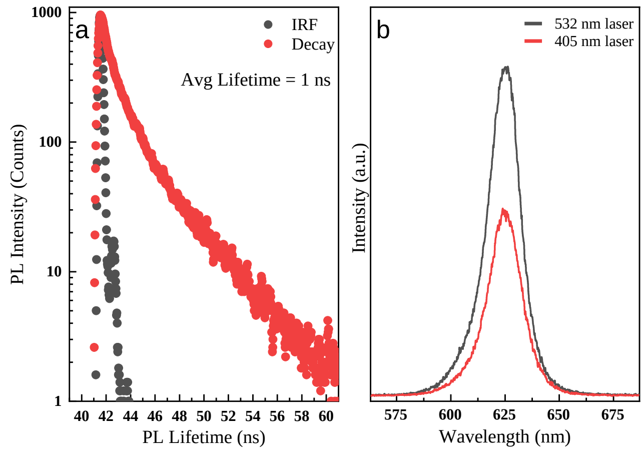

Figure S6. PL lifetime (a) and PL (b) of a bulk GaSe crystal.

Table S1. Central emission values of the PL emission obtained using the picosecond 405 nm laser as an excitation source.

Pressure	Central Emission (nm)	Central Emission (nm)	Central Emission (nm)	Central Emission (nm)	Central Emission (nm)
**Temp (°C)**	25°C	200°C	300°C	400°C	500°C
**0.5**	546	549	601	536	546
**1**	541	541	614	619	547
**2**	539	539	552	612	630
**3**	539	544	543	612	631
**5**	542	548	547	570	674

Table S2. FWHM values of the PL emission obtained using the picosecond 405 nm laser as an excitation source.

Pressure	FWHM (nm)	FWHM (nm)	FWHM (nm)	FWHM (nm)	FWHM (nm)
**Temp (°C)**	25°C	200°C	300°C	400°C	500°C
**0.5**	129	142.1	208	119	123
**1**	110	111	233	227	130
**2**	109	118	144	226	235
**3**	112	119	116	219	235
**5**	111	120	116	189	257

Supporting Information

Table S3. Central emission values of the PL emission obtained using the 532 nm continuous laser as an excitation source.

Pressure (T)	Central Emission (nm)	Central Emission (nm)	Central Emission (nm)	Central Emission (nm)	Central Emission (nm)
**Temp (°C)**	25°C	200°C	300°C	400°C	500°C
**0.5**	619	625	631	643	661
**1**	607	607	626	651	657
**2**	605	603	603	641	671
**3**	604	598	594	612	670
**5**	602	601	595	608	667

Table S4. FWHM values of the PL emission obtained using the 532 nm continuous laser as an excitation source.

Pressure (T)	FWHM (nm)	FWHM (nm)	FWHM (nm)	FWHM (nm)	FWHM (nm)
**Temp (°C)**	25°C	200°C	300°C	400°C	500°C
**0.5**	119	118	143	167	173
**1**	122	119	156	169	159
**2**	117	113	116	151	201
**3**	114	108	105	136	185
**5**	111	106	103	124	177

Table S5. Lifetime fitting parameters of the nanoparticles deposited at 0.5 torr.

	˚C	A	T1 (ns)	T2 (ns)	T3 (ns)	B1	B2	B3	Avg T (ns)
**0.5 Torr**	25	0.3765	0.731225	0.129307	3.627073	0.033445	0.205506	0.003596	0.264171
	200	0.30215	0.588688	3.41839	0.091332	0.032011	0.003651	0.290323	0.177437
	300	0.184215	0.078943	0.478933	2.884721	0.36545	0.024806	0.0011	0.112183
	400	0.465397	0.725004	4.066824	0.090214	0.027162	0.004158	0.298196	0.192724
	500	0.63646	0.410023	0.059662	2.369051	0.030851	0.442596	0.003301	0.098325

Table S6. Lifetime fitting parameters of the nanoparticles deposited at 1 torr.

	˚C	A	T1 (ns)	T2 (ns)	T3 (ns)	B1	B2	B3	Avg T (ns)
**1 Torr**	25	0.4274271	0.7540962	0.1585956	3.2313438	0.032112	0.1708399	0.0042126	0.313385
	200	0.3543137	0.7101392	4.3855397	0.1132297	0.0383503	0.0065988	0.2031747	0.31911
	300	0.2901749	0.5743158	0.1017695	2.5332397	0.0242911	0.289314	0.0017508	0.151668
	400	0.3237967	0.5250072	0.0906471	2.1481171	0.0330297	0.3004887	0.0032448	0.153074
	500	0.6234256	0.3056314	1.901737	0.0334506	0.0401633	0.0030453	0.9745709	0.0497813

Supporting Information

Table S7. Lifetime fitting parameters of the nanoparticles deposited at 2 torr.

	˚C	A	T1 (ns)	T2 (ns)	T3 (ns)	B1	B2	B3	Avg T (ns)
2 Torr	25	0.1750575	0.8592292	4.024655	0.1634982	0.0361958	0.0050445	0.1581142	0.387522
	200	0.4279814	0.6497374	0.1295084	3.0860115	0.040692	0.1824145	0.0052144	0.289746
	300	0.4761335	0.6633929	0.1328094	2.8366252	0.0391765	0.1749509	0.0059546	0.300413
	400	0.3829398	0.5212455	0.120366	1.9983458	0.0483285	0.1789817	0.0083602	0.269193
	500	0.3363721	0.4745001	0.0835505	2.0378402	0.0377126	0.3200647	0.0025176	0.138127

Table S8. Lifetime fitting parameters of the nanoparticles deposited at 3 torr.

	˚C	A	T1 (ns)	T2 (ns)	T3 (ns)	B1	B2	B3	Avg T (ns)
3 Torr	25	0.2446444	0.8186526	0.1679898	3.5211749	0.0320765	0.1575633	0.0032469	0.332638
	200	0.2543762	0.155196	0.7887412	3.6735355	0.1481988	0.0402304	0.0041208	0.362862
	300	0.3710518	0.7357355	0.1141849	4.3359261	0.0444039	0.1743323	0.0077847	0.38111
	400	0.4775388	0.7273778	2.7051167	0.1533387	0.0414428	0.0063572	0.1465101	0.359257
	500	0.5027549	0.4406089	2.1853672	0.0809183	0.0506979	0.0058303	0.2861599	0.169936

Table S9. Lifetime fitting parameters of the nanoparticles deposited at 5 torr.

	˚C	A	T1 (ns)	T2 (ns)	T3 (ns)	B1	B2	B3	Avg T (ns)
5 Torr	25	0.2624931	0.7419071	0.1670818	3.2860058	0.0396093	0.1460485	0.0054006	0.374413
	200	0.3642784	0.7089714	0.1346335	2.8893663	0.0364951	0.1959548	0.0036873	0.266413
	300	0.4108882	0.7462776	4.1229113	0.1276789	0.0438383	0.0076454	0.1691439	0.389039
	400	0.3218829	0.7118832	2.9545632	0.1211159	0.046988	0.0070916	0.1756727	0.329395
	500	0.2602637	0.0923414	0.5211756	1.8048778	0.2468006	0.0406756	0.0043775	0.177794
